# A Targeted “Next-Generation” Sequencing-Informatic Approach to Define Genetic Diversity in *Theileria orientalis* Populations within Individual Cattle: Proof-of-Principle

**DOI:** 10.3390/pathogens9060448

**Published:** 2020-06-05

**Authors:** Anson V. Koehler, Abdul Jabbar, Ross S. Hall, Robin B. Gasser

**Affiliations:** Department of Veterinary Biosciences, Melbourne Veterinary School, Faculty of Veterinary and Agricultural Sciences, The University of Melbourne, Parkville 3010, Australia; jabbara@unimelb.edu.au (A.J.); rossh@unimelb.edu.au (R.S.H.); robinbg@unimelb.edu.au (R.B.G.)

**Keywords:** *Theileria orientalis*, major piroplasm surface protein (*MPSP*) gene, multiplicity of infection (MOI), next-generation sequencing (NGS), SeekDeep

## Abstract

Oriental theileriosis is an economically important tickborne disease of bovines, caused by some members of the *Theileria orientalis* complex. Currently, 11 distinct operational taxonomic units (OTUs), or genotypes, are recognized based on their major piroplasm surface protein (*MPSP*) gene sequences. Two of these genotypes (i.e., *chitose* and *ikeda*) are recognized as pathogenic in cattle, causing significant disease in countries of the Asia-Pacific region. However, the true extent of genetic variation and associated virulence/pathogenicity within this complex is unknown. Here, we undertook a proof-of-principle study of a small panel of genomic DNAs (*n* = 13) from blood samples originating from individual cattle known to harbor *T. orientalis*, in order to assess the performance of a targeted “next-generation” sequencing-informatic approach to identify genotypes. Five genotypes (*chitose*, *ikeda*, *buffeli*, *type 4*, and *type 5*) were defined; multiple genotypes were found within individual samples, with dominant and minor sequence types representing most genotypes. This study indicates that this sequencing-informatic workflow could be useful to assess the nature and extent of genetic variation within and among populations of *T. orientalis* on a large scale, and to potentially employ panels of distinct gene markers for expanded molecular epidemiological investigations of socioeconomically important protistan pathogens more generally.

## 1. Introduction

Tickborne diseases (TBDs) substantially affect livestock in many parts of the world and have a major negative impact, particularly on resource-poor farming communities [1]. Key TBDs are caused by pathogens including *Anaplasma marginale* and *A. centrale* (anaplasmosis), *Babesia bovis, B. bigemina* and *B. divergens* (babesiosis), *Ehrlichia ruminantium* (cowdriosis/heartwater), as well as *Theileria annulata, T. parva*, and members of the *T. orientalis* complex (theileriosis), particularly in the tropics and subtropics [2].

Outbreaks of oriental theileriosis have had a significant, adverse economic impact on cattle farms in Australasia and parts of Asia [3,4,5,6,7,8]. The use of DNA-based diagnostic and analytical tools to establish the distribution and prevalence of distinct genetic variants (genotypes) within the *T. orientalis* complex has been central to the tracking and monitoring of oriental theileriosis outbreaks. Currently, 11 distinct *T. orientalis* genotypes, or operational taxonomic units (OTUs), including *chitose* (= *type* 1), *ikeda* (= *type* 2), *buffeli* (= *type* 3), *types* 4 to 8, and N1-N3, have been reported worldwide, and only the genotypes *chitose* and *ikeda* are recognized to be pathogenic in cattle [8,9].

Molecular assays utilized to detect, characterize, differentiate, or quantitate members of the *T. orientalis* complex in blood samples include the conventional polymerase chain reaction (PCR), loop-mediated isothermal amplification (LAMP), multiplex tandem PCR (MT-PCR), nested-PCR, reverse line blot hybridization assay (RLB), and quantitative PCR (qPCR) assays [8]. These methods use one or more genetic markers, such as the major piroplasm surface protein gene (*MPSP*), piroplasm membrane protein genes (*p23*, *p32* to *p34*), the small subunit of the nuclear ribosomal RNA gene (*SSU*), internal transcribed spacers (ITS) of nuclear ribosomal DNA, *β-tubulin* genes, and the mitochondrial cytochrome *c* oxidase subunit 3 gene (*cox*3) (reviewed by [10]).

*MPSP* has been most widely used in PCR to identify and differentiate genotypes in the *T. orientalis* complex [8]. In some cases, Sanger-sequencing of PCR products has been employed to define genotypes (cf. [8]). This approach has a disadvantage that only the predominant consensus sequence (with or without polymorphism) is recorded, but distinct sequence types within individual amplicons cannot be discerned. Although such sequence types can be defined by PCR-based cloning and sequencing [11], this approach is indirect (via molecular cloning), time-consuming, costly, and can introduce errors [12]. By contrast, the direct sequencing of PCR products using “deep”, short-read sequencing utilizing, for example, Illumina technology (www.illumina.com), could allow both sequence diversity within *T. orientalis* populations and infection intensity to be estimated.

Such a targeted “next-generation” (NGS) approach has been applied to *SSU* and other nuclear genetic markers to delineate a range of haemoprotozoan taxa and to explore genetic diversity within samples from individual host animals (e.g., [13,14,15,16,17]). However, for genetic markers that display marked sequence variation among genotypes, such as *MPSP*, there is an imperative to ensure that datasets produced are critically assessed for the presence of artefacts within amplicons (chimeras and jumping PCR errors) and in ensuing sequence data, preferably employing a bioinformatic workflow that is amenable to automation.

Recent studies of *Plasmodium* species (e.g., [18,19,20]) have demonstrated the exquisite sensitivity of targeted NGS, combined with the use of the sophisticated software, called SeekDeep v2.6.0 [21], to identify, record and quantitate, with confidence, distinct sequence types differing by as little as a point mutation within a PCR product. We expected that such a workflow could be applicable to and highly advantageous for the *T. orientalis* complex. Hence, we established, here, a proof-of-principle for a targeted NGS, i.e., a bioinformatic approach to reliably and directly discern genotypes within the *T. orientalis* complex using *MPSP* as the marker. This approach sets the scene for large-scale investigations of genetic variation in *Theileria* and a range of protistan taxa.

## 2. Results

### 2.1. Characteristics of Sequence Datasets, and Clustering of Reads to Define Genotypes

**Figure 1:**
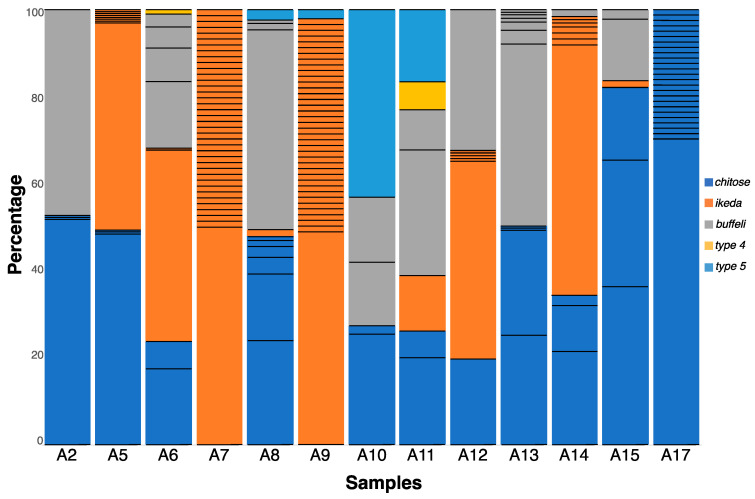
Genotypes of *Theileria orientalis* within individual samples, detected using the next-generation sequence-based bioinformatic approach, and presented as percentages of all *MPSP* sequence reads within individual samples. The numbers of thin, black, horizontal lines within individual bars (representing distinct nucleotide sequence types) define the multiplicity of infection (MOI) for individual samples (cf. Table 1).

A total of 219,238 “short reads” were generated from all *MPSP* amplicons. After processing with the SeekDeep algorithm, 19,439 reads were excluded, as they represented either singletons, chimeras, or were of poor quality, leaving 199,799 reads, and a mean count of 15,329 reads per sample (Table 1). Of the 13 samples, all but two (A7 and A17) contained mixed genotypes, and one sample (A11) contained all five genotypes identified in this study. The numbers of reads that clustered into individual genotypes were as follows: 76,955 (*chitose*), 52,053 (*ikeda*), 52,236 (*buffeli*), 1905 (*type 4*), and 16,650 (*type 5*) (Table 1 and Table S1); *chitose* was the commonest genotype, being identified in 11 of 13 samples; *ikeda* was recorded in nine samples; *type 5* in four samples; and *type 4* in two of the 13 samples (Table 1, Figure 1). Within genotype *chitose*, subgenotypes A and B (cf. [22]) were identified in six and eight samples, respectively.

### 2.2. Extensive MPSP Sequence Diversity within and among Samples

In total, 110 distinct *MPSP* sequences were identified in all 13 samples as follows: 37 for *chitose*, 58 for *ikeda*, 12 for *buffeli*, one for *type 4* and two for *type 5* (Table 1 and Table S1; Figure 1 and Figure S1). Of these 110 unique sequences, 72 (65.5%) were detected in a single sample (A17), whereas 38 (34.5%) were found in multiple samples. The number of different sequences, referred to as the “multiplicity of infection” (MOI) within a sample, ranged from four to 39 (Table 1 and Figure 1). Some samples contained one genotype, yet many sequences representing the same genotype reflected a higher MOI (e.g., with A7 containing as many as 38 sequence variants representing *ikeda*). Similarly, samples A9 and A17 had high MOI values (39 and 25, respectively, Figure 1), with little or no genotypic diversity (A9 contained *ikeda* and *type5*, whereas A17 contained only *chitose*), contrasting sample A2 which had the lowest MOI (= 4) (Table 1 and Figure 1).

When conceptually-translated amino acid sequences were compared with the dominant (reference) sequence representing each of the five genotypes, 85 of the 110 amino acid sequences were unique. Therefore, most mutations were non-synonymous and were single point mutations (SNPs). Sequences representing each *buffeli* and *chitose* B differed at multiple nucleotide positions, whereas those representing each *ikeda* and *chitose* A varied only at single positions. There was only one sequence variant (representing *ikeda*) whose reading frame was disrupted by a premature stop codon (cf. Figure S1).

### 2.3. Genotypes within Samples Usually Represented by a Unique, Dominant Nucleotide Sequence

Individual genotypes within individual samples were mostly represented by a dominant “majority” sequence and, typically, sequences differed from this dominant sequence by only one or two nucleotides (Figure S1). For example, 38 sequence variants were identified in Sample A7, with a dominant sequence equating to 57% of 8167 reads, while the next commonest sequence was represented by 3.7% of all reads (Figure 1). For this sample, which contained solely the genotype *ikeda*, there was only a point mutation difference between the dominant sequence and all other sequence types. Likewise, sequences representing *chitose* A differed by single point mutations, whereas some representing *chitose* B and *buffeli* varied at multiple positions (Figure S1). For the 11 samples containing *chitose*, coinfections of subgenotypes A and B were detected in three samples, type A was found exclusively in three samples, and type B was found exclusively in five samples (Table 1). Of all 110 sequence variants, most (*n* = 97) were novel, meaning that they did not have an identical match to any published sequence available in the GenBank database.

## 3. Discussion

Here, we established proof-of-principle for a targeted NGS, i.e., a bioinformatic approach to reliably and directly discern genotypes within the *T. orientalis* complex using a portion of the *MPSP* gene as the marker. Using this approach, we simultaneously detected five genotypes of the *T. orientalis* complex and, surprisingly, a total of 110 distinct sequence variants in all samples analyzed. As SeekDeep has been shown to eliminate artefactual sequences and retain sequence variants with very high confidence [21], we were able to infer the multiplicity of infection (MOI) of particular genotypes. The findings revealed that mixed-genotypic infections were common, in spite of the small sample size tested in this study. The ability to reliably discern sequence types, and attribute them to particular populations should prove useful for comprehensive investigations of genetic variation within species of *Theileria*. The ability to associate sequence types and genotypes with clinical and asymptomatic cases would facilitate the identification and tracking of sources of infection, and the monitoring of outbreaks (cf. [9]).

NGS has been applied previously to *Theileria*, but in a manner distinct from this proof-of-principle study. Recently, a whole genome sequencing (WGS) method has been used to explore the *T. orientalis* complex in cattle [23], but such an approach is not presently scalable for routine monitoring or diagnostic purposes. Another WGS method was employed to investigate hemoparasites in ticks [24], but the amount of data produced was excessive and the procedure was costly as compared with targeted NGS which used one or a few loci to detect infections [25]. Targeted NGS approaches have been applied to selected *SSU* markers, resulting in the detection of 14 taxa of *Babesia* and *Theileria* in ruminants [14], *Theileria* species in African antelopes [26] and buffaloes [16,17], and *Hepatozoon*, *Babesia*, and *Trypanosoma* species in dogs [15]. However, the *SSU* region does not provide the resolution required for assessing genetic variation within taxa. An NGS approach with some similarity to ours has been applied previously to *Plasmodium falciparum* using regions of genes that encode the merozoite surface protein (*MSP*) [19,20], the *P. falciparum* apical membrane antigen 1 (*pfama1* [18,27]) and genome-wide SNPs (reviewed by [28]). In one study [21], the authors employed targeted NGS of multiple proposed vaccine candidate- and drug resistance-genes, along with mock microbiome datasets.

The gene encoding *MPSP*, originally described as a 32 kDa immunogenic protein with a promise to be a vaccine candidate [29], is orthologous to the *p32* gene of *T. parva* and the *Tams1* gene of *T. annulata*, which are both known for having a mosaic pattern of antigenic diversity [9,30]. Accordingly, the high levels of sequence (genetic) variation found within and among some samples in this study (Table 1 and Figure 1) had been assumed, but its extent was not appreciated based on published information for *T. orientalis*. Interestingly, Hemmink et al. [13] examined allelic variation for six antigen-encoding genes of *T. parva* using a targeted NGS method and found a substantial number of gene variants within blood samples from some cattle investigated, indicating multiple infection event(s) in these animals. Similarly, in our study, the detection of up to five distinct genotypes in some samples studied here (e.g., A11, Figure 1) suggests that multiple infection events had occurred via suitable tick vectors or a single transmission of multiple genotypes by a tick [13]. We hypothesize that this marked variation reflects antigenic drift or shift in the pathogen(s) to evade or subvert immunological attack by the host animal. This hypothesis warrants testing. Ectopic recombination, as reported for the “*var*” genes of *P. falciparum* (see [31,32,33]) could be an alternative possibility, although such recombination could result in marked chromosomal rearrangement and organismal destruction [34].

## 4. Materials and Methods

Blood samples (*n* = 13) from cattle (*Bos taurus* or *Bos indicus*) were available from previously published studies, approved by the Research and Ethics Committee of the College of Veterinary and Animal Sciences, University of Bahawalpur, Pakistan [35] and the Ethiopian National Research Ethics Review Committee [36]. These blood samples were known to contain *T. orientalis* DNA based on results of conventional PCR testing [35,36].

Genomic DNAs were extracted from 200 µL of each of the whole-blood samples by sodium-dodecyl sulphate (SDS)-proteinase K digestion and mini-column purification using a commercial kit (DNeasy^®^ Blood & Tissue, Qiagen, Hilden, Germany), following the manufacturer’s instructions, and samples were stored at −20 °C until further analysis. Then, a 344 bp region of the *MPSP* gene was amplified from individual samples using primers MPSP-AJ-F (5-TTC ACT CCA ACA GTC GCC CAC A-3) and MPSP-AJ-R1 (5-ACG TAA ACT TTG ACT GCG GTG-3) [37] in 50 µL containing 10 mM Tris-HCl (pH 8.4), 50 mM KCl (Promega, Madison, WI, USA), 3.5 mM MgCl_2_, 6.25 M of each deoxynucleotide triphosphate (dNTP), 100 pmol of each primer, and 1 U of *GoTaq* polymerase (Promega, Madison, WI, USA). The PCR cycling conditions were as follows: an initial denaturation at 95 °C for 5 min, followed by 35 cycles at 95 °C for 30 s, 58 °C for 30 s, 72 °C for 1 min, followed by 72 °C for 5 min. No-DNA (negative) and known positive (*T. orientalis*) control samples were included in each PCR run. PCR products were examined following agarose (1.5%) gel electrophoresis, and then subjected to Illumina sequencing on a MiSeq platform at Novogene (Beijing, China; (en.novogene.com) using the established protocol from Illumina. Paired-end sequence data (consensus length 301 bp) from which adaptors were trimmed were provided in Fastq files. Using SeekDeep v2.6.0 software [21], with settings designed specifically for Illumina data, the “clusters” algorithm was used to remove singletons, low quality reads, and chimeras. Then, sequences were clustered using the default cut-off (0.5%) for a fraction of all reads within a cluster to minimize errors. The “process clusters” algorithm was used to combine the reads from all amplicons. The, unique sequences were converted into the FASTA file format. Values for the multiplicity of infection (MOI, the number of distinct sequences within an individual sample) and nucleotide diversity among all samples were calculated using DnaSP v5 [38]. All sequence data were deposited in the GenBank database (accession nos. MT428433 to MT428542).

## 5. Conclusions

In this study, first, we elected to critically assess the sequencing and informatic approach to convince ourselves of the reliability of the datasets and the ability of the SeekDeep algorithm to eliminate PCR-induced artefacts from the datasets. Our results indicate that this approach is useful and has the potential to be applied to a large panel of distinct genetic markers, preferably derived from well-assembled genomes. We believe that a focus on using multiple divergent single-copy genes would be advantageous, circumventing problems associated with in vitro recombination of paralogous sequences (representing multigene families) during PCR. We envisage that the use of a multiplex PCR-based NGS-informatic workflow, employing replicates to ensure repeatability and reproducibility and to gain further confidence in MOI data, should provide a unique platform for future molecular epidemiological surveys and population genetic analyses of protists of veterinary and medical importance.

## Figures and Tables

**Table 1:** List of bovine blood DNA samples tested for *Theileria orientalis* using the next-generation sequencing-based bioinformatic approach, and the total number of *MPSP* sequence reads and multiplicity of infection (MOI, i.e., the number of distinct sequence types) in each sample.

Sample Code	Total No. of Reads (MOI)	Genotype					
		*Chitose A*	*Chitose B*	*Ikeda*	*Buffeli*	*Type 4*	*Type 5*
A2	18,573 (4)	MT428436			MT428452		
A5	19,215 (11)	MT428436		MT428433			
A6	20,523 (9)		MT428435	MT428433	MT428453	MT428454	
A7	8167 (38)			MT428433			
A8	14,038 (11)	MT428436	MT428435	MT428495	MT428434		MT428479
A9	8957 (39)			MT428433			MT428439
A10	26,800 (5)		MT428435		MT428434		MT428439
A11	27,608 (7)	MT428436	MT428435	MT428433	MT428434	MT428454	MT428439
A12	14,171 (7)		MT428435	MT428433	MT428434		
A13	10,032 (11)		MT428435		MT428434		
A14	8813 (11)		MT428440	MT428433	MT428452		
A15	13,332 (6)	MT428436	MT428435	MT428433	MT428434		
A17	9570 (25)	MT428436					

## Supplementary Materials

The following are available online at https://www.mdpi.com/2076-0817/9/6/448/s1, Figure S1: Nucleotide alignments of the sequence variants for the *Theileria orientalis* complex (including genotypes *buffeli*, *chitose* (A & B), and *ikeda*), produced by targeted NGS using a region of the major piroplasm surface protein gene (*MPSP*). Alignments are compared to the most frequent sequence variant for each genotype. Synonymous nucleotide changes are in bold, and nonsynonymous amino acid changes are highlighted in colour. Genotypes *type 4* and *type 5* were omitted here, as only three sequences were recorded, Table S1: Codes and GenBank accession numbers of all 110 (partial) *MPSP* sequences obtained from 13 bovine blood DNA samples tested for *Theileria orientalis* using next-generation sequencing-based bioinformatic approach. Total number of the sequence reads for individual genotypes in samples are listed.

## References

[1] Makala L.H., Mangani P., Fujisaki K., Nagasawa H. (2003). The current status of major tick-borne diseases in Zambia. Vet. Res..

[2] Jabbar A., Abbas T., Sandhu Z.U., Saddiqi H.A., Qamar M.F., Gasser R.B. (2015). Tick-borne diseases of bovines in Pakistan: Major scope for future research and improved control. Parasit Vectors.

[3] Kamau J., de Vos A.J., Playford M., Salim B., Kinyanjui P., Sugimoto C. (2011). Emergence of new types of *Theileria orientalis* in Australian cattle and possible cause of theileriosis outbreaks. Parasit Vectors.

[4] Perera P.K., Gasser R.B., Anderson G.A., Jeffers M., Bell C.M., Jabbar A. (2013). Epidemiological survey following oriental theileriosis outbreaks in Victoria, Australia, on selected cattle farms. Vet. Parasitol..

[5] Perera P.K., Gasser R.B., Firestone S.M., Anderson G.A., Malmo J., Davis G., Beggs D.S., Jabbar A. (2014). Oriental theileriosis in dairy cows causes a significant milk production loss. Parasit Vectors.

[6] Watts J.G., Playford M.C., Hickey K.L. (2016). *Theileria orientalis*: A review. N. Z. Vet. J..

[7] Gebrekidan H., Nelson L., Smith G., Gasser R.B., Jabbar A. (2017). An outbreak of oriental theileriosis in dairy cattle imported to Vietnam from Australia. Parasitology.

[8] Gebrekidan H., Perera P.K., Ghafar A., Abbas T., Gasser R.B., Jabbar A. (2020). An appraisal of oriental theileriosis and the *Theileria orientalis* complex, with an emphasis on diagnosis and genetic characterisation. Parasitol. Res..

[9] Sivakumar T., Hayashida K., Sugimoto C., Yokoyama N. (2014). Evolution and genetic diversity of *Theileria*. Infect. Genet. Evol..

[10] Mans B.J., Pienaar R., Latif A.A. (2015). A review of *Theileria* diagnostics and epidemiology. Int. J. Parasitol. Parasit Wildl..

[11] Perera P.K., Gasser R.B., Firestone S.M., Smith L., Roeber F., Jabbar A. (2015). Semiquantitative multiplexed tandem PCR for detection and differentiation of four *Theileria orientalis* genotypes in cattle. J. Clin. Microbiol..

[12] Gasser R.B. (2006). Molecular tools—Advances, opportunities and prospects. Vet. Parasitol..

[13] Hemmink J.D., Sitt T., Pelle R., de Klerk-Lorist L.M., Shiels B., Toye P.G., Morrison W.I., Weir W. (2018). Ancient diversity and geographical sub-structuring in African buffalo *Theileria parva* populations revealed through metagenetic analysis of antigen-encoding loci. Int. J. Parasitol..

[14] Chaudhry U., Ali Q., Rashid I., Shabbir M.Z., Ijaz M., Abbas M., Evans M., Ashraf K., Morrison I., Morrison L. (2019). Development of a deep amplicon sequencing method to determine the species composition of piroplasm haemoprotozoa. Ticks Tick Borne Dis..

[15] Huggins L.G., Koehler A.V., Ng-Nguyen D., Wilcox S., Schunack B., Inpankaew T., Traub R.J. (2019). A novel metabarcoding diagnostic tool to explore protozoan haemoparasite diversity in mammals: A proof-of-concept study using canines from the tropics. Sci. Rep..

[16] Combrink L., Glidden C.K., Beechler B.R., Charleston B., Koehler A.V., Sisson D., Gasser R.B., Jabbar A., Jolles A.E. (2020). Age of first infection across a range of parasite taxa in a wild mammalian population. Biol. Lett..

[17] Glidden C.K., Koehler A.V., Hall R.S., Saeed M.A., Coppo M., Beechler B.R., Charleston B., Gasser R.B., Jolles A.E., Jabbar A. (2019). Elucidating cryptic dynamics of *Theileria* communities in African buffalo using a high-throughput sequencing informatics approach. Ecol. Evol..

[18] Miller R.H., Hathaway N.J., Kharabora O., Mwandagalirwa K., Tshefu A., Meshnick S.R., Taylor S.M., Juliano J.J., Stewart V.A., Bailey J.A. (2017). A deep sequencing approach to estimate *Plasmodium falciparum* complexity of infection (COI) and explore apical membrane antigen 1 diversity. Malar. J..

[19] Zhong D., Lo E., Wang X., Yewhalaw D., Zhou G., Atieli H.E., Githeko A., Hemming-Schroeder E., Lee M.C., Afrane Y. (2018). Multiplicity and molecular epidemiology of *Plasmodium vivax* and *Plasmodium falciparum* infections in east Africa. Malar. J..

[20] Metoh T.N., Chen J.H., Fon-Gah P., Zhou X., Moyou-Somo R., Zhou X.N. (2020). Genetic diversity of *Plasmodium falciparum* and genetic profile in children affected by uncomplicated malaria in Cameroon. Malar. J..

[21] Hathaway N.J., Parobek C.M., Juliano J.J., Bailey J.A. (2017). SeekDeep: Single-base resolution de novo clustering for amplicon deep sequencing. Nucleic Acids Res..

[22] Jenkins C., Micallef M., Alex S.M., Collins D., Djordjevic S.P., Bogema D.R. (2015). Temporal dynamics and subpopulation analysis of *Theileria orientalis* genotypes in cattle. Infect. Genet. Evol..

[23] Bogema D.R., Micallef M.L., Liu M., Padula M.P., Djordjevic S.P., Darling A.E., Jenkins C. (2018). Analysis of *Theileria orientalis* draft genome sequences reveals potential species-level divergence of the ikeda, chitose and buffeli genotypes. BMC Genom..

[24] Brinkmann A., Hekimoglu O., Dincer E., Hagedorn P., Nitsche A., Ergunay K. (2019). A cross-sectional screening by next-generation sequencing reveals *Rickettsia*, *Coxiella*, *Francisella*, *Borrelia*, *Babesia*, *Theileria* and *Hemolivia* species in ticks from Anatolia. Parasit Vectors.

[25] Goodwin S., McPherson J.D., McCombie W.R. (2016). Coming of age: Ten years of next-generation sequencing technologies. Nat. Rev. Genet..

[26] Pienaar R., Josemans A., Latif A.A., Mans B.J. (2020). The host-specificity of *Theileria* sp. (sable) and *Theileria* sp. (sable-like) in African bovidae and detection of novel *Theileria* in antelope and giraffe. Parasitology.

[27] Boyce R.M., Hathaway N., Fulton T., Reyes R., Matte M., Ntaro M., Mulogo E., Waltmann A., Bailey J.A., Siedner M.J. (2018). Reuse of malaria rapid diagnostic tests for amplicon deep sequencing to estimate *Plasmodium falciparum* transmission intensity in western Uganda. Sci. Rep..

[28] Koepfli C., Mueller I. (2017). Malaria epidemiology at the clone level. Trends Parasitol..

[29] Kobayashi N., Onuma M., Kirisawa R., Ohgitani T., Takahashi K., Sasaki N., Kawakami Y. (1987). Monoclonal antibodies against intraerythrocytic merozoites (piroplasms) of *Theileria sergenti*. Nihon Juigaku Zasshi.

[30] Gubbels M.J., Katzer F., Hide G., Jongejan F., Shiels B.R. (2000). Generation of a mosaic pattern of diversity in the major merozoite-piroplasm surface antigen of *Theileria annulata*. Mol. Biochem. Parasitol..

[31] Freitas-Junior L.H., Bottius E., Pirrit L.A., Deitsch K.W., Scheidig C., Guinet F., Nehrbass U., Wellems T.E., Scherf A. (2000). Frequent ectopic recombination of virulence factor genes in telomeric chromosome clusters of *P. falciparum*. Nature.

[32] Duffy M.F., Byrne T.J., Carret C., Ivens A., Brown G.V. (2009). Ectopic recombination of a malaria *var* gene during mitosis associated with an altered *var* switch rate. J. Mol. Biol..

[33] Claessens A., Hamilton W.L., Kekre M., Otto T.D., Faizullabhoy A., Rayner J.C., Kwiatkowski D. (2014). Generation of antigenic diversity in *Plasmodium falciparum* by structured rearrangement of *var* genes during mitosis. PLoS Genet..

[34] Montgomery E., Charlesworth B., Langley C.H. (1987). A test for the role of natural selection in the stabilization of transposable element copy number in a population of *Drosophila melanogaster*. Genet. Res..

[35] Gebrekidan H., Abbas T., Wajid M., Ali A., Gasser R.B., Jabbar A. (2017). Molecular characterisation of *Theileria orientalis* in imported and native bovines from Pakistan. Infect. Genet. Evol..

[36] Gebrekidan H., Gasser R.B., Baneth G., Yasur-Landau D., Nachum-Biala Y., Hailu A., Jabbar A. (2016). Molecular characterization of *Theileria orientalis* from cattle in Ethiopia. Ticks Tick Borne Dis..

[37] Cufos N., Jabbar A., de Carvalho L.M., Gasser R.B. (2012). Mutation scanning-based analysis of *Theileria orientalis* populations in cattle following an outbreak. Electrophoresis.

[38] Librado P., Rozas J. (2009). DnaSP v5: A software for comprehensive analysis of DNA polymorphism data. Bioinformatics.

