# Supplementary material for: A Targeted “Next-Generation” Sequencing-Informatic Approach to Define Genetic Diversity in Theileria orientalis Populations within Individual Cattle: Proof-of-Principle
Source: Pathogens. 2020 Jun 5;9(6):448. doi: 10.3390/pathogens9060448 (PMC7350381; doi:10.3390/pathogens9060448)
Supplement: Supplementary file 1 [file pathogens-09-00448-s001.zip › Figure S1.pdf]

Figure S1. Nucleotide alignments of the sequence variants for the *Theileria orientalis* complex (including genotypes: *buffeli*, *chitose* (A & B) and *ikeda*), produced by targeted NGS using a region of the major piroplasm surface protein gene (*MPSP*). Alignments are compared to the most frequent sequence variant for each genotype. Synonymous nucleotide changes are in bold, and nonsynonymous amino acid changes are highlighted in colour. Genotypes *type 4* and *type 5* were omitted here, as only three sequences were recorded.

buffeli

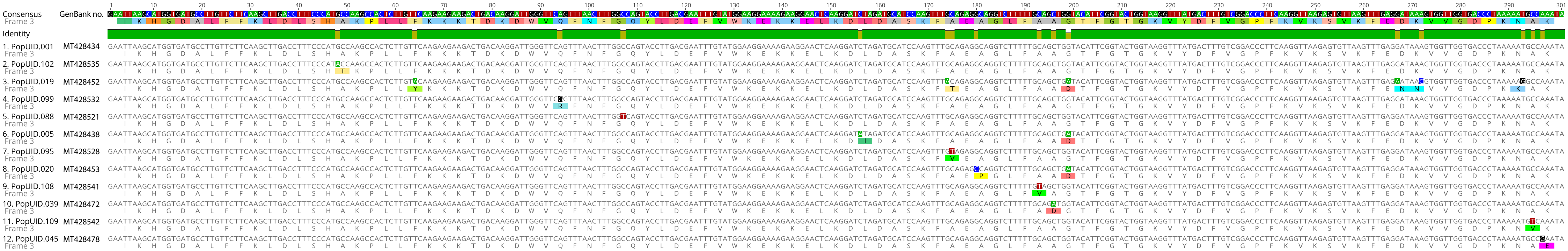

***chitose* A**

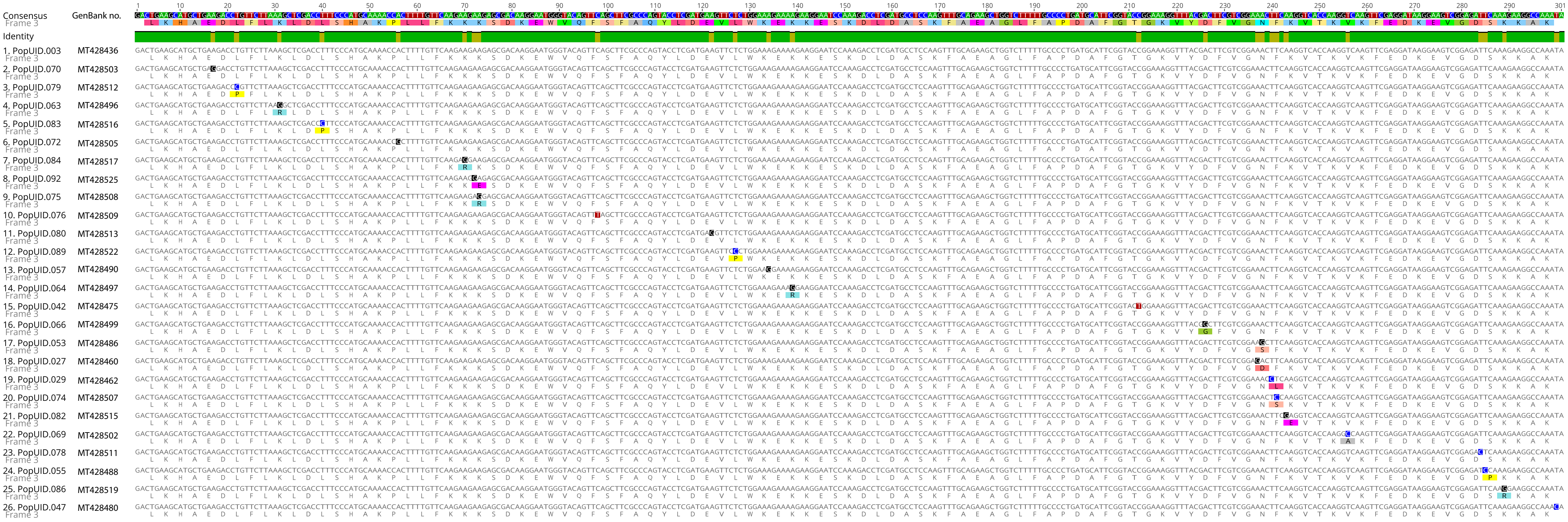
Consensus
Frame 3
GenBank no.
Identity
1. PopUID.003 MT428436
2. PopUID.070 MT428503
3. PopUID.079 MT428512
4. PopUID.063 MT428496
5. PopUID.083 MT428516
6. PopUID.072 MT428505
7. PopUID.084 MT428517
8. PopUID.092 MT428525
9. PopUID.075 MT428508
10. PopUID.076 MT428509
11. PopUID.080 MT428513
12. PopUID.089 MT428522
13. PopUID.057 MT428490
14. PopUID.064 MT428497
15. PopUID.042 MT428475
16. PopUID.066 MT428499
17. PopUID.053 MT428486
18. PopUID.027 MT428460
19. PopUID.029 MT428462
20. PopUID.074 MT428507
21. PopUID.082 MT428515
22. PopUID.069 MT428502
23. PopUID.078 MT428511
24. PopUID.055 MT428488
25. PopUID.086 MT428519
26. PopUID.047 MT428480

***chitose* B**

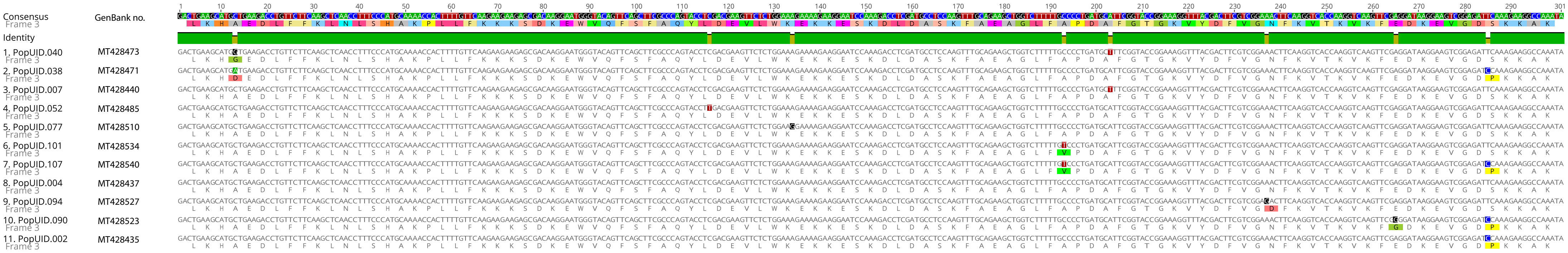
Consensus
Frame 3
GenBank no.
Identity
1. PopUID.040 MT428473
2. PopUID.038 MT428471
3. PopUID.007 MT428440
4. PopUID.052 MT428485
5. PopUID.077 MT428510
6. PopUID.101 MT428534
7. PopUID.107 MT428540
8. PopUID.004 MT428437
9. PopUID.094 MT428527
10. PopUID.090 MT428523
11. PopUID.002 MT428435

ikeda

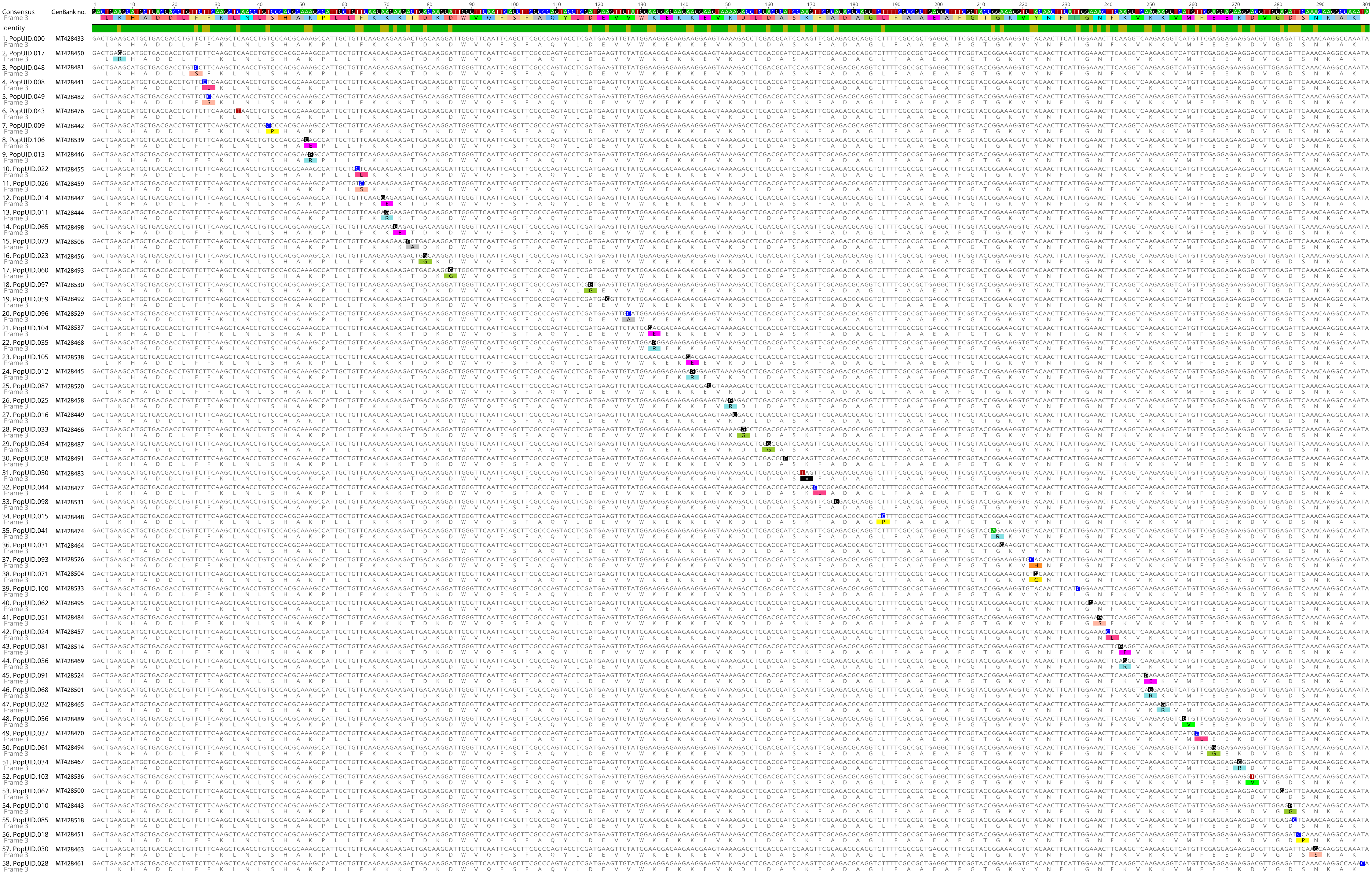

Consensus
GenBank no.
Identity
1. PopUID.000 MT428433
2. PopUID.017 MT428450
3. PopUID.048 MT428481
4. PopUID.008 MT428441
5. PopUID.049 MT428482
6. PopUID.043 MT428476
7. PopUID.009 MT428442
8. PopUID.106 MT428539
9. PopUID.013 MT428446
10. PopUID.022 MT428455
11. PopUID.026 MT428459
12. PopUID.014 MT428447
13. PopUID.011 MT428444
14. PopUID.065 MT428498
15. PopUID.073 MT428506
16. PopUID.023 MT428456
17. PopUID.060 MT428493
18. PopUID.097 MT428530
19. PopUID.059 MT428492
20. PopUID.096 MT428529
21. PopUID.104 MT428537
22. PopUID.035 MT428468
23. PopUID.105 MT428538
24. PopUID.012 MT428445
25. PopUID.087 MT428520
26. PopUID.025 MT428458
27. PopUID.016 MT428449
28. PopUID.033 MT428466
29. PopUID.054 MT428487
30. PopUID.058 MT428491
31. PopUID.050 MT428483
32. PopUID.044 MT428477
33. PopUID.098 MT428531
34. PopUID.015 MT428448
35. PopUID.041 MT428474
36. PopUID.031 MT428464
37. PopUID.093 MT428526
38. PopUID.071 MT428504
39. PopUID.100 MT428533
40. PopUID.062 MT428495
41. PopUID.051 MT428484
42. PopUID.024 MT428457
43. PopUID.081 MT428514
44. PopUID.036 MT428469
45. PopUID.091 MT428524
46. PopUID.068 MT428501
47. PopUID.032 MT428465
48. PopUID.056 MT428489
49. PopUID.037 MT428470
50. PopUID.061 MT428494
51. PopUID.034 MT428467
52. PopUID.103 MT428536
53. PopUID.067 MT428500
54. PopUID.010 MT428443
55. PopUID.085 MT428518
56. PopUID.018 MT428451
57. PopUID.030 MT428463
58. PopUID.028 MT428461
